# Perinatal Lethal Gaucher Disease due to RecNcil Recombinant Mutation in the* GBA* Gene Presenting with Hydrops Fetalis and Severe Congenital Anemia

**DOI:** 10.1155/2018/2549451

**Published:** 2018-05-09

**Authors:** Ekta Bhutada, Timothy Pyragius, Scott G. Petersen, Frans Niemann, Admire Matsika

**Affiliations:** ^1^Mater Pathology, Mater Health Services, South Brisbane, QLD, Australia; ^2^Department of Biomedical Sciences, Faculty of Medicine, University of Queensland, Brisbane, QLD, Australia; ^3^National Referral Laboratory, Genetics and Molecular Pathology, SA Pathology, North Adelaide, SA, Australia; ^4^Centre for Maternal Fetal Medicine, Mater Health Services, South Brisbane, QLD, Australia

## Abstract

A 35-year-old woman presented at 27-week gestation with hypertension and pedal edema. Antenatal scan showed hydrops fetalis and growth restriction. Cordocentesis showed severe fetal anemia. This was treated with multiple in utero blood transfusions with no clinically significant improvement and intrauterine death occurred at 28 weeks. Perinatal autopsy confirmed severe hydrops with hepatosplenomegaly and visceral effusions. Microscopic examination of the reticuloendothelial organs showed widespread infiltration by large mono- and multinucleate histiocytic cells with fibrillary appearance (“Gaucher cells”). DNA extracted from fetal tissue was submitted for analysis by next generation sequencing which revealed homozygosity for the RecNcil mutation in the* GBA* gene. Both parents were found to be heterozygous for the variant. The case report highlights a severe form of Gaucher disease with histopathological and molecular confirmation that presents with hydrops fetalis and severe refractory anemia. It also emphasizes the importance of perinatal autopsy coupled with exome sequencing in confirming syndromic diagnosis in the modern area.

## 1. Introduction

Perinatal lethal Gaucher disease (PLGD) is a rarer variant of type 2 Gaucher disease (GD). The multisystemic nature and varied presentations of the disease makes it a challenge to diagnose in early pregnancy [1]. A correct and timely diagnosis of GD is crucial for appropriate management, genetic counselling, and future family planning. We describe a case of PLGD presenting prenatally with thrombocytopenia, transfusion-refractory severe anemia, and non-immune fetal hydrops. These clinical features were initially suspected to be due to a primary bone marrow disorder.

## 2. Case Report

A 35-year-old woman in her third pregnancy was referred to our tertiary institution at 27-week gestation for investigation of unexplained fetal hydrops. She had originally presented to her family practitioner with bilateral pedal edema and hypertension. Her obstetric history is significant for one previous uninvestigated male stillbirth followed by an uncomplicated pregnancy. She has chronic hypertension treated with labetalol. The woman and her partner are both of East Indian ethnic background and there is no history of consanguinity.

Obstetric ultrasound assessment showed severe fetal hydrops with increased abdominal circumference due to ascites and elevated Middle Cerebral Artery Peak Systolic Velocity. Subsequent amniocentesis and maternal blood tests excluded common chromosomal and infectious causes of fetal anemia. Cordocentesis confirmed fetal anemia (hemoglobin level 46 g/dL) and thrombocytopenia (platelet count: 9 × 10^9^/L). The white cell count was normal (13.5 × 10^9^/L). Two intrauterine blood transfusions were administered in an attempt to improve fetal hemoglobin levels. However, no significant improvement in the anemia or hydrops was observed. Intrauterine fetal death occurred at 28 weeks and 3 days.

Parental consent for partial autopsy examination that excluded the brain was obtained. Post mortem weight of 1650 g (normal range 1144.3 ± 163.8 g) was higher than expected at 28 weeks and is attributable to the anasarca. The abdominal circumference was also increased and all other external fetal measurements were within normal range for stated gestational age. External examination showed subtle facial anomalies including a high arched palate with no clefting, flat broad nose with hypertelorism, and rounded face. Elbow and knee joints were stiff with fixed flexion deformities and pterygia on the flexor surfaces. There was no collodion or ichthyotic changes of the edematous skin. Internal autopsy examination showed 30 mls of pleural fluid bilaterally together with smaller amounts of pericardial and peritoneal effusions. The lungs and kidney weights were below the 5th percentile for gestational age with a lung: body weight ratio of 0.01 (pulmonary hypoplasia). The liver was average sized at 64.0 g and the spleen was enlarged 18.4 g (1.32–4.10 g). All other internal organ weights were within interquartile range for 28-week gestation.

**Figure 1:**
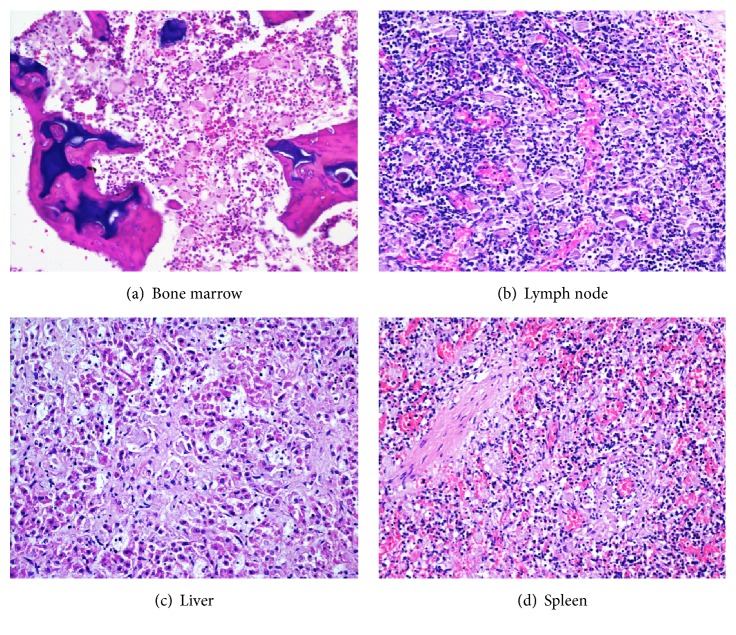
Sections of the bone marrow, lymph node, liver, and spleen showing large mononuclear and multinuclear histiocytic cells with fine fibrillary appearance (“Gaucher cells”) (H&E ×200).

Microscopic examination of the liver, spleen, bone marrow, and intra-abdominal lymph nodes showed infiltration by mono- and multinuclear histiocytic cells containing ample cytoplasm with delicate fibrillary appearance (Figure 1). The cells displaced native hematopoietic cells in these organs and were CD68 positive on immunohistochemistry. The intracytoplasmic material was PAS-D and luxol fast blue (LFB) positive. Alcian blue was negative. Attempt at electron microscopy was unsuccessful due to marked post mortem degenerative change of the Gaucher cells. Histopathological sections of the skin showed subcutaneous oedema. Light microscopy of other internal organs examined was normal for stated gestational age.

**Figure 2:**
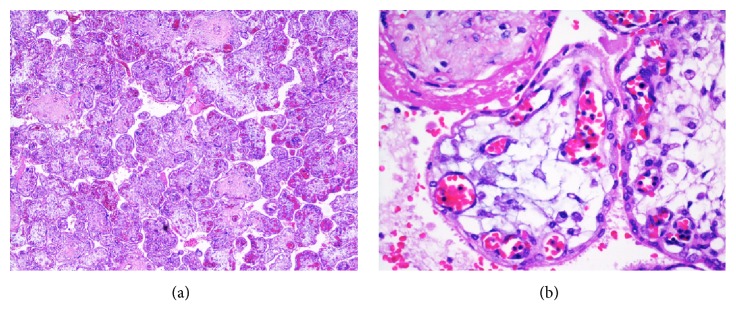
Placental section at low power shows hydropic villi with increased numbers of nucleated red blood cells in the fetal vessels (H&E ×100). (b) High power view shows histiocytic cells with fibrillary appearance in the villous stroma (H&E ×400).

Placental examination showed placentomegaly with villous hydrops, fetal erythroblastosis, and infiltration of villous stroma by aforementioned histiocytic cells (Figure 2). Hypertrophic decidual vasculopathy, attributable to the chronic maternal hypertension, was also demonstrated. Post mortem skeletal survey excluded skeletal dysplasia, microbiology studies were negative, and cytogenetics analysis using Affymetrix CytoScan™ 750 kb array showed a normal male karyotype with no copy number aberration.

Fetal DNA extracted from cord blood, fetal liver, thymus, and spleen, together with parental DNA, was submitted. Targeted exome sequencing of 88 genes associated with fetal hydrops and lysosomal storage disorders was performed. Next generation sequence analysis was performed using the 4813 gene Illumina TruSight One™ panel on the Illumina NextSeq (R) Sequencing System™. Variant calls were analysed using Variant Studio v2.2. Variants with MAF > 1% in population databases and variants found previously in unrelated individuals were excluded from the analysis. Interpretation of variant pathogenicity was based on the American College of Medical Genetics and Genomics (ACMG) and Association for Clinical Genetic Science (ACGS) guidelines.

This analysis revealed that the fetus was homozygous for the RecNcil recombinant mutation ([c.1448T>C;1483G>C;1497G>C]) in the* GBA* (glucocerebrosidase) gene. Sanger sequencing was used to confirm the presence of this pathogenic variant and testing of the parents showed that both were heterozygous for the same variant. A diagnosis of Gaucher disease was confirmed on correlation with autopsy findings. Testing of the parents showed that both were heterozygous for the same variant.

## 3. Discussion

Gaucher disease (GD) is the most common lysosomal storage disorder seen worldwide. It was first described by Philippe Gaucher in 1882 and is now known to result from deficiency of lysosomal glucocerebrosidase enzyme [2]. The* GBA* gene is located on chromosome 1q21 and more than 300 variants of the gene have been reported as pathogenic to date. Deficiency of this enzyme leads to accumulation of glucosylceramide and other glycolipids in the lysosomes of macrophages of the reticuloendothelial system and other organs. According to the Gaucher registry, the prevalence of GD is 1/57000 to 1/75000 births worldwide, 1/4000 in the USA, and 1/57000 in Australia. The disease is more prevalent in Ashkenazi Jews in whom the incidence is 1/800 births.

GD is an autosomal recessive disorder with subtyping based on clinical features and natural course of the disease. Type 1 GD is the most common and does not have neurologic involvement; GD2 is the acute neuronopathic form; and GD3 is the chronic neuronopathic form. Type 2 GD is the most severe and progressive form, manifesting either prenatally or in the first months of life. It comprises about 1% of all GD cases and shows no ethnic predilection. Perinatal forms are exceptionally rare. While type 2 GD is generally associated with certain mutations in the glucocerebrosidase gene, there is also significant genotypic heterogeneity and genotype-phenotype correlation is not completely understood [3].

Type 2 GD, or acute neuronopathic GD, presents with neurological involvement within the first months of life and progresses rapidly, culminating in severe degeneration and death in infancy or early childhood. Perinatal-lethal GD (PLGD), as in our case, is considered a variant of type 2 GD. Perinatal presentation is often with non-immune hydrops fetalis, in utero fetal demise or neonatal cardiorespiratory distress. Fetal hydrops is a poor prognostic indicator with such pregnancies culminating in premature delivery, in utero or neonatal demise [4].

Mignot et al. [1] have reported the RecNcil mutation to be the most frequent genetic aberration in PLGD cases reported to date. Patients homozygous for the ([c.1448T>C;1483G>C;1497G>C]) RecNcil recombinant allele tend to have a severe phenotype, often with neurologic features. The RecNcil mutation is due to a recombination event with the highly homologous and adjacent GBAP1 pseudogene. This results in the common p.Leu483Pro (c.1448T>C) being coallelic with both the p.Ala495Pro (c.1483G>C) and p.Val499Val (c.1497G>C) sequence variants. Because of the pseudogene, this variant can easily be missed by testing on next generation sequencing platforms for the disease.

The confirmation of an index case of GD allows for appropriate genetic counselling and testing of other family members. As unusual mechanisms including de novo mutations have been described in patients with GD, the recurrence risk is not always obvious [5]. In this case, as both the parents are heterozygous for GD, there is a significant risk (25%) of the disease affecting a future offspring. Knowledge of the specific variant involved in a family also enables early antenatal diagnostic testing in future pregnancy.

This case highlights the utility of perinatal autopsy examination coupled with exome sequencing in the current genomic era for investigation of perinatal deaths. Preautopsy clinical impression was that of a primary hemopoietic disorder. The possibility of Gaucher disease as an underlying cause was first raised after histopathological examination of the spleen, liver, and bone marrow sections. Genetic testing alone without a full phenotype is unlikely to have arrived at the correct diagnosis, given the presence of the RecNcil mutation and large number of variants of uncertain significance often found with “blinded” exome sequencing of preterm fetal deaths. We have recently reported on the diagnostic challenges and role of whole genome sequencing on the next generation platform in perinatal death investigations [6].

In conclusion, as one of the commonest lysosomal storage disorder, GD must be excluded in all cases of unexplained non-immune hydrops fetalis. We have shown that PLGD due to RecNcil mutation results in a severe, fatal form of hydrops with arthrogryposis and congenital anemia that is refractory to intrauterine blood transfusions. This finding has important clinical management implications for fetomaternal practitioners who may attempt to treat such patients with in utero blood transfusions. A multidisciplinary approach to perinatal autopsy that incorporates histopathology, radiology, and exome sequencing in the investigation of unexplained stillbirths still has a role to play in the modern genomic era of medicine [7].
